# Germination Behavior and Early Seedling Growth in *Abies pinsapo* Boiss. Seeds

**DOI:** 10.3390/plants11202715

**Published:** 2022-10-14

**Authors:** María Victoria Bravo-Navas, Carolina Sánchez-Romero

**Affiliations:** Departamento Botánica y Fisiología Vegetal, Universidad de Málaga, Campus de Teatinos s/n., 29071 Málaga, Spain

**Keywords:** *Abies pinsapo*, light response, seed germination, seedling growth, temperature response

## Abstract

*Abies pinsapo* Boiss. is a conifer endemic to southern Spain. It is categorized as an endangered species in the IUCN list and the plant communities it forms are considered unique ecosystems, being the remains of fir forests occupying the Mediterranean basin during the last glaciations. Understanding seed germination and plant production is essential for the management and conservation of *A. pinsapo* stands. The objective of this work was to investigate the effect of temperature and light on germination behavior and early seedling growth of *A. pinsapo* seeds from different populations. The results obtained reveal a significant influence of seed origin on germination percentage. Temperature played a critical role on germination rate, with optimal results at 15 °C. Light only significantly affected germination in seeds from Grazalema, although significant population × light and temperature x light interactions could be inferred. In relation to germination kinetics, different responses to the environmental factors tested were observed among seeds from different provenances. Globally, the temperature influenced all the germination parameters, except germination onset. However, light affected germination initiation and speed. Early seedling growth depended on seed origin and temperature. The temperature played a determinant role as temperatures above 15 °C strongly limited plantlets development. Light only significantly influenced root length in plantlets obtained from Grazalema seeds.

## 1. Introduction

*Abies pinsapo* Boiss. is a fir species endemic to southern Spain. It belongs to the circum-Mediterranean firs, which covered large areas during the last ice-age periods [1,2]. However, along the Neogene-Quaternary, the loss of Pinaceae biodiversity and extinctions occurred, and in the Pleistocene, climatic fluctuations severely reduced the size of *A. pinsapo* populations [3,4]. Currently, Spanish fir is considered as a Tertiary relict species [5]. It inhabits ecological and geographical restricted areas, occupying shaded locations with a northerly or occasionally easterly or northeasterly exposure along the western Baetic Range [4,6].

Currently, *A. pinsapo* is present in three main enclaves: (1) Sierra de las Nieves and surrounding areas presents the most important stands, with isolated groves in the ranges of Alcor, Caparaín, Real, Istán, Río Verde and Gialda; (2) the population of Los Reales de Sierra Bermeja, which is divided into separated groves due to repeated fires; and (3) the population of Sierra de Grazalema and surrounding areas. Groves and isolated stands are also found in the western part of Monte Prieto, the sides of El Montón and on the northern slopes of Zafalgar and Los Pinos [7,8].

*A. pinsapo* stands are subjected to multiple threats. Traditionally, they have suffered a high anthropogenic pressure due to activities such as clearance for agriculture, livestock raising, illegal felling, intensive grazing, arson and the use of their timber for construction, firewood or charcoal making. Besides, *A. pinsapo* is exposed to pests such as *Dioryctria aulloi*, *Cryphalus numidicus*, *Mindarus abietinus, Cinara pectinatae* and *Cinara confinis*, and fungus such as *Armillaria mellea* and *Heterobasidion annosum* [8]. Moreover, due to its ecological requirements, *A. pinsapo* is especially sensitive to warming and reduced precipitations associated to climate change [8]. These threats along with its fragmented and very limited distribution range make *A. pinsapo* a species highly vulnerable [5]. In fact, *A. pinsapo* is listed in different regional, national and international catalogues, including the International Union for Conservation of Nature’s Red List of Threatened Species, where it is classified as endangered under criteria B1ab(i,ii,iii)+2ab(i,ii,iii) [9].

Moreover, *A. pinsapo* forests constitute one of the most unique ecosystems in the Iberian Peninsula due to their relict nature [2]. These plant formations are included in the NATURA 2000 Network and in the Habitats Directive (Council Directive 92/43/EEC of 21 May 1992 on the conservation of natural habitats and of wild fauna and flora). According to their ecological importance and vulnerability, the three enclaves where *A. pinsapo* is present are covered by different forms of protection, and a plan for *A. pinsapo* recovery was implemented [10].

The production of plants for reforestation and the realization of germination and propagation experiences are some of the measures for conserving and managing *A. pinsapo* stands. The availability of optimized seed germination protocols, ensuring acceptable germination rates and the obtainment of healthy and vigorous plantlets is, therefore, very important for *A. pinsapo* conservation.

Germination is a very important phase in the life cycle of a plant, since it is the first step for the establishment of a new generation [11]. Due to the extreme fragility of the emergent seedling, germination is a high-risk moment [12]. Hence, germination timing has an adaptive character [13], with germination patterns trying to find environmental conditions suitable for seedling establishment [14]. In this context, environmental signals play an essential role by contributing to detect proper gap conditions [15]. The mechanisms involved in environmental signals transduction would be under a strong selective pressure, and natural selection will shape particular patterns of environmental sensitivity for each plant species, dependent on its past history [14].

Seed germination percentages of *Abies* species are generally low due to different problems [16]. In *A. pinsapo*, some studies on germination have been carried out using seeds collected from forest and isolated trees [17] and sown in different habitats [18]. However, the influence of specific environmental factors has not been investigated.

The aim of this work was to investigate the germination behavior of *A. pinsapo* seeds from different populations, examining their response to temperature and light conditions simulating natural habitats. Apart from the final germination percentage, germination kinetics was also studied by determining parameters related to germination timing, rate and synchrony. The quality of the plantlets obtained was evaluated as well, owing to the determinant influence of early seedling growth on final tree recruitment in both natural habitats and plant nurseries.

This investigation would help to understand the ecophysiology of *A. pinsapo* germination and to predict the effects of climate change on natural populations. Additionally, it contributes to the optimization of germination protocols, thus improving seed management and plant production for both in situ and ex situ conservation.

## 2. Results

### 2.1. Seed Viability

Seed viability did not significantly vary among the populations tested. Slightly lower values were obtained in seeds from Los Reales, with 43.33%. In the rest of seed provenances, viability percentages ranged from 63.33 to 73.33% (Grazalema: 63.33%; Ronda: 70%; Sierra Real: 70%; Yunquera: 73.33%).

### 2.2. Seed Germination

**Figure 1:**
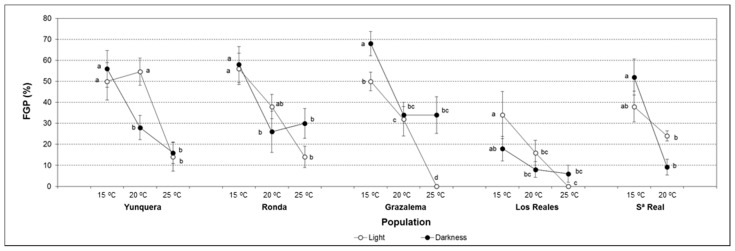
The effect of temperature and light on FGP of *A. pinsapo* seeds from different provenances. Data represent the mean ± SEM. Different letters within each population indicate significant differences by the LSD test with a significance level of 0.05.

Germination percentage under different temperature and light conditions significantly varied depending on the seed origin (Table S1), with maximum final germination percentages (FGP) ranging from 34% in seeds from Los Reales to 68% in seeds from Grazalema (Figure 1).

As revealed by three-way ANOVA, seed germinability was significantly affected by the temperature (Table S1), with a similar significant effect in all populations (Table S2). In general, optimum results were achieved at 15 °C. Incubation at higher temperatures significantly decreased FGP.

A light effect was only evident in seeds from Grazalema, and a significant population × light interaction was inferred (Tables S1 and S2). Contrarily to the rest of the populations, in which light conditions slightly improved or did not influence FGP, in seeds from Grazalema, higher germination percentages were obtained in darkness. As revealed by the significant temperature × light interaction (Table S1), the light effect depended on the temperature. In general, whereas at 15 °C slightly higher germination was obtained in darkness; at 20 °C, better results were achieved under light conditions. At 25 °C, the light exerted a negative effect, leading to lower germination rates and, in some cases, to the complete inhibition of the process. Although a similar behavior could be observed in all populations, the temperature × light interaction only was statistically significant in seeds from Grazalema and Sierra Real (Table S2).

### 2.3. Germination Kinetics

The pattern of seed germination was significantly influenced by seed origin and the environmental factors tested (Table S1).

**Figure 2:**
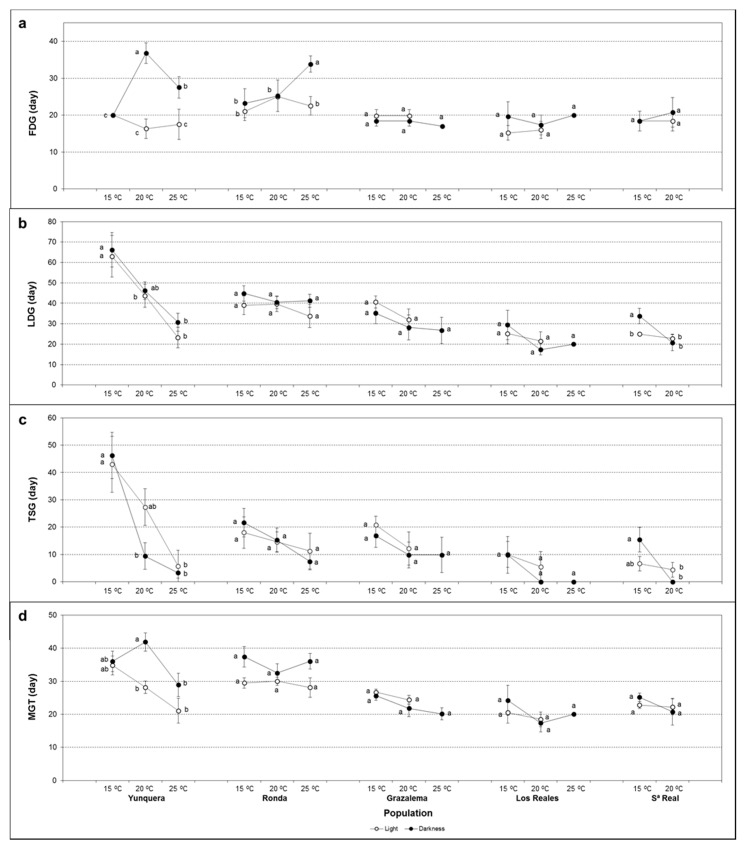
Effect of temperature and light on germination parameters of *A. pinsapo* seeds from different provenances: (**a**) FDG; (**b**) LDG; (**c**) TSG; and (**d**) MGT. Data represent the mean ± SEM. Different letters within each population indicate significant differences by the LSD test with a significance level of 0.05.

The first day of germination (FDG) ranged from 18 to 25 days, depending on seed provenance (Figure 2a and Table S1). Contrarily to the rest of germination parameters assessed, in general, FDG was not affected by the temperature (Table S1), despite the fact that a significant effect was found in seeds from Yunquera (Table S2). Although globally light significantly reduced the time required for germination initiation (Table S1), its effect was significantly influenced by the seed origin, as revealed by the significant population × light interaction (Table S1). Thus, although the effect of light was only statistically significant in seeds from Yunquera (Table S2), the same trend was observed in all populations, except Grazalema, in which slightly lower FDG values were attained in darkness.

The last day of germination (LDG) was influenced by the population, the temperature, and the interaction between both factors (Figure 2b and Table S1). Globally, higher temperatures brought forward the day on which the last germination event occurred, although this effect only was significant in seeds from Yunquera and Sierra Real (Table S2).

The time spread of germination (TSG) also varied depending on the population, the temperature and the interaction population × temperature (Figure 2c and Table S1). In general, germination synchronization increased at higher temperatures. Despite the same trend being observed in all populations, similarly to LDG, the temperature effect was only statistically significant in seeds from Yunquera and Sierra Real (Table S2).

Germination speed was significantly affected by all the predictor variables (Table S1), although the effect of temperature was only statistically significant in seeds from Yunquera and light only influenced mean germination time (MGT) in seeds from Yunquera and Ronda (Table S2). Globally, lower MGT, i.e., higher speeds, were obtained at light and as the temperature increased, especially from 15 to 20 °C (Figure 2d).

Therefore, different responses to temperature and light were appreciated among populations. While germination kinetics was strongly influenced by these environmental factors in Yunquera seeds, no parameters were affected in seeds from Grazalema and Los Reales. In Ronda and Sierra Real, only specific aspects of germination dynamics were significantly influenced by temperature or light.

In general, correlation analysis revealed moderate associations between FGP and both LDG and TSG, i.e., higher germination percentages were associated to late germination completion and reduced synchronization (Table S3). A weak correlation between FGP and MGT was also observed (Table S3), thus showing an association between slower germination and higher germination percentages. Most of these associations were found in the different populations, with the exception of Grazalema, in which no correlation could be inferred between FGP and the rest of the germination parameters (Table S4).

### 2.4. Early Seedling Growth

**Figure 3:**
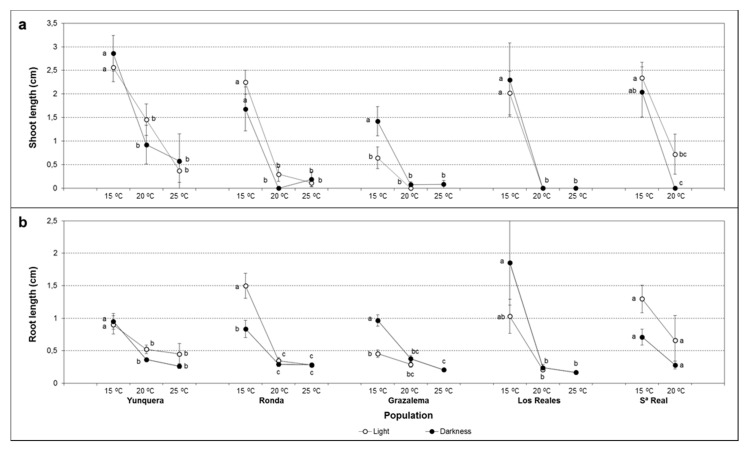
Effect of temperature and light on (**a**) shoot and (**b**) root length of seedlings obtained from *A. pinsapo* seeds from different provenances. Data represent the mean ± SEM. Different letters within each population indicate significant differences by the LSD test with a significance level of 0.05.

In relation to the plantlets obtained, significantly different shoot lengths were obtained depending on seed origin (Figure 3a and Table S5). However, no significant differences due to the population were observed in root growth (Figure 3b and Table S5).

The temperature exerted a determinant influence on early seedling development, significantly affecting shoot and root length (Table S5). Plants with longer shoots and roots were obtained at 15 °C, although no significant differences were observed between 20 and 25 °C. The same effect was evident in all populations, except in seeds from Sierra Real, in which a significant influence of the temperature on root length could not be inferred (Table S6).

Globally, root length was also affected by the interactions population × temperature, population × light, and population × temperature × light (Table S5). Although a similar trend was observed in general, as revealed by two-way ANOVA, the interaction between temperature and light was only statistically significant in seeds from Ronda and Grazalema (Table S6). Interestingly, Grazalema was the only population in which light significantly influenced root growth. In seeds from this provenance, significantly longer roots were obtained in darkness compared to light conditions at 15 °C.

**Figure 4:**
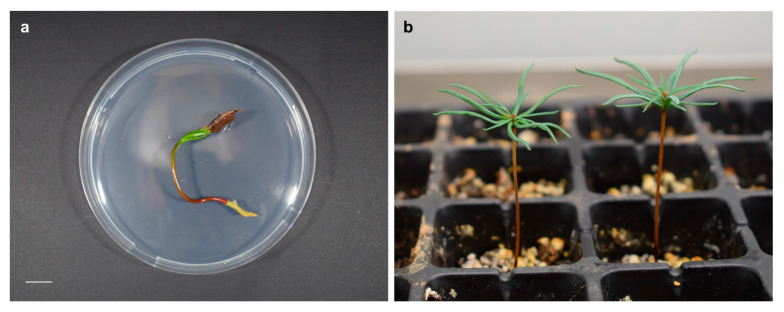
Seedlings obtained from *A. pinsapo* seeds germinated at 15 °C and under light conditions (**a**) at the end of the experiment and (**b**) four months after transference to ex vitro conditions. Bar = 1 cm.

Germination environmental conditions also affected plant quality. Seedlings obtained in darkness were thinner and weaker due to etiolation, whereas plants developed under light conditions presented good aspect, with robust, brown shoots and well-developed roots. Better aspect was in general observed in plants obtained at 15 °C due to its greater development (Figure 4a). Plant growth adequately progressed after transference to ex vitro conditions (Figure 4b).

## 3. Discussion

The temperature was a critical factor in *A. pinsapo* germination. The germination response to temperature was similar in all populations, with optimal results at 15 °C and a sharp decline at higher temperatures. Stimulation of seed germination at low temperatures is a common feature in many woody Mediterranean species and is considered an adaptive trait in plants from this region [19]. In conifers, higher germination at 15–20 °C was reported in multiple species of *Pinus* [19,20,21,22,23,24]. A sharp germination decline at temperatures above the optimum has also been reported in *Picea engelmannii* [25,26] and *Pinus bungeana* [21]. According to Guo et al. [21], the germination decrease at warm temperatures could be due to thermo-inhibition or thermo-dormancy, a phenomenon of secondary dormancy [27].

A relevant role in seed germination has usually been attributed to light [28]. In conifers, a stimulating effect of light has been reported in *Pinus sylvestris* [29], *Pinus nigra* [30], *Pinus brutia* [23], *Pinus halepensis* [23,24], *Pinus canariensis* [19], *Widdringtonia whytei* [31] and *Abies fraseri* [32]. In *A. pinsapo*, a direct effect of light was not observed, as also reported in *Pinus halepensis* [19], *Pinus pinaster* [19], *Pinus pinea, Pinus nigra* [20] and *Pinus bungeana* [21]. Nevertheless, a significant temperature × light interaction was found, thereby revealing a variable influence of light depending on the germination temperature. This interaction, although with different temperature effects, was also described in *Picea abies* [33], *Abies cephalonica* [34] and *Pinus sylvestris* [19]. As reported in *Pinus brutia* [23], light broadened the germination temperature. Thus, while at 20 °C a slight germination decline was observed under light conditions, a sharp decrease was found in darkness. As proposed by Ahola and Leinonen [33] in *Picea abies*, a light-sensing mechanism regulating germination in a temperature-dependent way might have evolved in *A. pinsapo*. Nevertheless, while in *Picea abies* this mechanism could control germination under cool conditions, in *A. pinsapo* it would operate above the optimum temperature (15 °C).

As revealed by a significant light × population interaction, the effect of light also depended on seed provenance. Contrarily to the rest of the populations in which no light effect was observed, significantly higher germination rates were achieved in darkness in seeds from Grazalema. A differential light effect depending on the seed origin was also found in *Chamaecyparis thyoides* [35], *Pinus canariensis* and *Pinus halepensis* [19]. Therefore, although in conifer seeds light sensitivity appears to be determined by the species [21], intraspecific variations can also be expected, especially in species with a wide geographical distribution [19].

Significant differences in seed germination were observed among *A. pinsapo* populations. Interpopulation variability in germination is considered a general trend in conifer species [19]. Thus, intraspecific differences have been reported in *Abies guatemalensis* [36], *Abies koreana* [37], *Pinus nigra* [19,22], *Pinus pinea*, *Pinus pinaster* [19], *Chamaecyparis thyoides* [35], *Pinus flexilis* and *Picea engelmannii* [26]. Differences in germination capacity among seeds from different provenances have been attributed to genetic variations [25,38], although an important influence of the maternal environment has also been pointed out [39]. Soil characteristics such as nutrient availability can affect seed vigor, as found in *Picea mariana* [38]. Furthermore, climate aspects such as the temperature in the seed’s developmental environment can affect gene expression via genetic and epigenetic mechanisms, and determine subsequent life-history traits [40]. Nevertheless, in *Abies koreana*, variations in germination ability were explained by differences in seed viability, as a significant positive correlation between the percentages of germination and of sound seeds was found [37]. Although no significant differences in seed viability were observed in *A. pinsapo* seeds from different provenances, lower viability values were obtained in seeds from Los Reales, the population in which lower germination rates were attained.

Germination kinetics is a relevant feature due to its implications in seed management and plant regeneration. In *A. pinsapo*, the germination pattern was influenced by both environmental factors and seed origin.

Earlier germination can facilitate seedling establishment, as it results in a longer growing season, which allows the formation of plants with an extensive root system, appropriate to successfully overcome the summer stress conditions [15,26].

A temperature effect on germination initiation has been repeatedly described in conifers, such as *Abies lasiocarpa* [25], *Picea engelmanii* [25,26], *Araucaria angustifolia* [41] and *Pinus flexilis* [26]. Although higher temperatures normally reduced time to germination [26], a direct, general temperature effect was not found in *A. pinsapo*. Significant differences in FDG among temperature treatments could only be observed in seeds from Yunquera. However, globally, light advanced germination onset. A similar light effect was reported in *Abies fraseri* [32].

Germination synchronization was affected by the temperature, with a significant reduction of TSG as the temperature increased. Although the same trend was in general observed, the same variations due to seed origin were evident, as revealed by the statistically significant population × temperature interaction. Different to temperature, light did not influence the time required to complete germination, as previously reported in *Pinus bungeana* [21].

Germination speed is an important trait, as rapid germination may provide seedlings more time to grow under favorable moisture conditions [42]. In *A. pinsapo*, seeds from different populations germinated at different speeds. Interpopulation differences in this germination feature were also found in *Abies amabilis* [43]. Similarly to germination capacity, this germination trait was under a strong genetic control [43]. Globally, temperature and light also affected MGT, although these effects were only significant in seeds from Yunquera and Ronda. As previously found in *Abies fraseri* [44] and *Pinus nigra* [22]), warm temperatures accelerated germination. Higher velocities were also recorded under light conditions, although in *Pinus bungeana* light did not influence germination speed [21].

Apart from the germination percentage, the quality of the seedlings obtained is also essential for plant regeneration. As previously reported in *Abies guatemalensis* [36], differences in growth performance were observed in seeds from different provenances, although in *A. pinsapo* this effect was restricted to shoot development. According to Liesebach et al., certain habitat, climatic and topographic differences among the locations of origin could explain variability in the seedling traits [45]. The temperature had a determinant effect on seedling growth, as temperatures above 15 °C strongly limited the development of both shoots and roots.

Differently to shoots, the effect of the environmental factors on root growth depended on seed origin, as revealed by the significant population × temperature and population × light interactions. Grazalema was the only population in which light affected root development, with longer roots obtained in darkness.

In conclusion, the environmental factors tested and seed origin influenced both germination behavior and seedling growth. The temperature played an essential role in determining both processes. According to the results obtained, changes in the weather pattern caused by climate change can have detrimental effects on *A. pinsapo* populations. The negative influence of warmer temperatures on seed germination and seedling growth would lead to a decrease in tree recruitment in their natural habitats. Populations decline, migration to higher altitudes or risks for long-term persistence could, therefore, occur under a climate change scenario.

## 4. Materials and Methods

### 4.1. Plant Material

Mature cones of *Abies pinsapo* Boiss. were collected in five natural populations, covering the range of distribution of the species: Sierra de Grazalema Natural Park, Los Reales de Sierra Bermeja Natural Area and three locations in Sierra de las Nieves National Park, due to the larger extension of this area: Pinsapar de Ronda, Sierra de Yunquera and Sierra Real (Table 1). The climate of the study region is Mediterranean, and classified as Csb using the Köppen classification system [46], with a mean annual temperature of 12.2–14.2 °C and a mean annual precipitation of 822–1145 mm (except Sierra de Grazalema, with 1894 mm) [47].

Scales were removed from cones and seeds were carefully separated from scales. A pool of seeds was obtained for each population including all seeds extracted from cones, except undeveloped, damaged or insect-infected seeds, which were manually removed. Seeds were stored at 15 °C in darkness for a maximum of two months.

Cones collection were carried out with the necessary authorizations according to the Spanish fir Recovery Plan (Consejería de Agricultura, Ganadería, Pesca y Desarrollo Sostenible, Junta de Andalucía, Spain) [10].

### 4.2. Seed Viability

In order to examine seed quality of the seed lots, seed viability was assessed by using the tetrazolium test, a biochemical assay in which living tissue changes the uncolored 2,3,5-triphenyl-tetrazoliumchloride (TTC) to the insoluble red 1,3,5-triphenylformazan, due to the activity of dehydrogenase enzymes involved in respiration. In non-living tissues, TTC remains uncolored [48].

Vital staining was carried out according to Witty [49]. Seeds were bisected along their longitudinal axes with the aid of a scalpel. Seed sections were incubated in an aqueous solution of TTC at 0.5% (*w*/*v*) for 2 h at 37 °C and darkness. Red stained seeds were considered as viable. Three replicates with 10 seeds each were examined per population.

### 4.3. Germination Experiments

In order to investigate the influence of the environmental factors temperature and light on germination, seeds from different populations were subjected to different temperature and light treatments in a factorial design experiment.

Seeds were surface-sterilized by immersion in a 0.5% (*v*/*v*) sodium hypochlorite solution supplemented with Tween 20 (1 drop/100 mL) for 10 min and rinsed three times for 5 min with sterile distilled water. Disinfected seeds were placed, under aseptic conditions, onto the culture medium in Petri dishes (90 × 15 mm). The culture medium consisted of distilled water gelled with 6 g L^−1^ agar. Media sterilization was carried out by autoclaving for 20 min at 121 °C and 0.1 MPa. Seeds were incubated at 15, 20 and 25 °C under both darkness and light conditions (16 h light/8 h dark photoperiod and 40 μmol m^−2^ s^−1^ irradiance level, provided by cool-white fluorescent lamps). Five replicates, with ten seeds each, were performed per seed provenance and treatment.

### 4.4. Data Taken and Statistical Analysis

Germination data were recorded weekly. Seeds were considered as germinated when shoot and/or radicle elongation was ≥1.5 mm. The experiment was considered as completed when no additional germination events were observed for 4 weeks in any of the treatments.

Treatment effects were assessed in terms of germination rate and kinetics. For this purpose, the following germination parameters were calculated from the cumulative germination curve of each germination replicate [50]:

- FGP: Total number of germinated seeds divided by the total number of seeds used in the replication × 100.

- FDG: Day on which the first germination event occurred, i.e., lag time before germination onset.

- LDG: Day on which the last germination event occurred.

- TSG: An estimation of germination synchronization, calculated as the time elapsed between the first and last germination events occurring in a seed replicate.

- MGT [51]: An index of germination speed, calculated by using the following equation:

(1) $$MGT=\frac{\sum_{i=1}^{k}ni\;ti}{N}$$

where, *ni* is the number of seeds germinated between scoring intervals, *ti* is the number of days from the beginning of the test, and *N* is the total number of seeds germinated in the treatment at the end of experiment.

The influence of treatments on early seedling growth was evaluated by measuring shoot and root length of plantlets obtained at the end of the experiment.

Data of viability percentage were analyzed by one-way ANOVA. Data of the germination parameters and seedling growth were analyzed by three-way ANOVA in order to elucidate the effects of the variables temperature, light and population, as well as their corresponding interactions. When a significant effect of the population was found, the influence of the temperature and light was separately analyzed in seeds from the different provenances. Following ANOVA, data were subjected to the least significant difference (LSD) test in order to determine the possible existence of significant differences among treatments [52].

Correlation between the determined germination parameters was estimated by the Pearson coefficient.

The significance level was 0.05 in all cases. Statistical analyses were performed using SPSS 25.0 (IBM SPSS Statistics, Armonk, NY, USA).

## Figures and Tables

**Table 1:** Details of the source populations of *Abies pinsapo* seeds.

Location	Latitude	Longitude	Altitude (m.a.s.l.)
Pinsapar de Ronda	36°41′37′′ N	5°07′20′′ W	1300–1700
Sierra de Yunquera	36°43′51′′ N	4°57′11′′ W	1000–1500
Sierra Real	36°37′13′′ N	4°58′41′′ W	1000–1449
Sierra de Grazalema	36°46′11′′ N	5°24′03′′ W	1000–1400
Los Reales de Sierra Bermeja	36°29′29′′ N	5°12′02′′ W	1100–1449

m.a.s.l.: meters above sea level.

## Data Availability

Not applicable.

## Supplementary Materials

The following supporting information can be downloaded at: https://www.mdpi.com/article/10.3390/plants11202715/s1, Table S1: Significance by three-way ANOVA of single and combined effects of the population, temperature and light for the germination parameters determined in *Abies pinsapo* seeds; Table S2: Significance by two-way ANOVA of single and combined effects of the temperature and light for the germination parameters determined in *Abies pinsapo* seeds from different provenances; Table S3: Results of Pearson correlation analysis between different germination parameters of *Abies pinsapo* seeds from different provenance, Table S4: Results of Pearson correlation analysis between FGP and the rest of germination parameters determined in *Abies pinsapo* seeds from different provenances; Table S5: Significance by three-way ANOVA of single and combined effects of the population, temperature and light for shoot and root length of seedlings obtained from *Abies pinsapo* seeds; Table S6: Significance by two-way ANOVA of single and combined effects of the temperature and light for shoot and root length of seedlings obtained from *Abies pinsapo* seeds from different provenances.
