# Supplementary material for: Germination Behavior and Early Seedling Growth in Abies pinsapo Boiss. Seeds
Source: Plants (Basel). 2022 Oct 14;11(20):2715. doi: 10.3390/plants11202715 (PMC9611577; doi:10.3390/plants11202715)
Supplement: Supplementary file 1 [file plants-11-02715-s001.zip › plants-1931335-supplementary.pdf]

Table S1. Significance by three-way ANOVA of single and combined effects of the population, temperature and light for the germination parameters determined in *Abies pinsapo* seeds.

Predictor variable	FGP	FDG	LDG	TSG	MGT
Population (P)	**0.000**	**0.000**	**0.000**	**0.000**	**0.000**
Temperature (T)	**0.000**	0.196	**0.000**	**0.000**	**0.002**
Light (L)	0.337	**0.000**	0.316	0.407	**0.005**
P x T	0.189	0.151	**0.006**	**0.006**	0.131
P x L	**0.011**	**0.046**	0.778	0.814	0.067
T x L	**0.000**	0.092	0.501	0.249	0.857
P x T x L	0.494	**0.004**	0.963	0.619	0.103

Significant *P* values are in bold ($P < 0.05$).

Table S2. Significance by two-way ANOVA of single and combined effects of the temperature and light for the germination parameters determined in *Abies pinsapo* seeds from different provenances.

Predictor variable	FGP	FDG	LDG	TSG	MGT
**Yunquera**					
Temperature (T)	**0.000**	**0.033**	**0.000**	**0.000**	**0.003**
Light (L)	0.295	**0.000**	0.450	0.344	**0.004**
T x L	0.057	**0.001**	0.934	0.308	0.102
**Ronda**					
Temperature (T)	**0.000**	0.122	0.550	0.118	0.656
Light (L)	0.746	0.061	0.166	0.974	**0.005**
T x L	0.195	0.146	0.712	0.743	0.433
**Grazalema**					
Temperature (T)	**0.000**	0.720	0.282	0.287	0.058
Light (L)	**0.001**	0.329	0.399	0.537	0.276
T x L	**0.041**	1.000	0.882	0.877	0.656
**Los Reales**					
Temperature (T)	**0.004**	0.928	0.366	0.368	0.461
Light (L)	0.243	0.353	0.998	0.611	0.724
T x L	0.214	0.615	0.474	0.636	0.501
**Sierra Real**					
Temperature (T)	**0.000**	0.700	**0.017**	**0.014**	0.287
Light (L)	0.945	0.700	0.252	0.496	0.839
T x L	**0.026**	0.700	0.075	0.054	0.421

Significant *P* values are in bold ($P < 0.05$).

Table S3. Results of Pearson correlation analysis between different germination parameters determined in *Abies pinsapo* seeds.

Parameters		FGP	FDG	LDG	TSG	MGT
**FGP**	Pearson r	1				
	P value					
**FDG**	Pearson r	-0.145	1			
	P value	0.115				
**LDG**	Pearson r	0.486	0.257	1		
	P value	**0.000**	**0.005**			
**TSG**	Pearson r	0.560	-0.199	0.896	**1**	
	P value	**0.000**	**0.030**	**0.000**		

MGT	Pearson r	0.326	0.672	0.802	0.505	1
	P value	**0.000**	**0.000**	**0.000**	**0.000**	

Significant *P* values are in bold ($P < 0.05$).

Table S4. Results of Pearson correlation analysis between FGP and the rest of germination parameters determined in *Abies pinsapo* seeds from different provenances.

Population	FGP	FDG	LDG	TSG	MGT
**FGP Yunquera**					
Pearson r	1	-0.393	0.599	0.701	0.256
P value		**0.035**	**0.001**	**0.000**	0.180
**FGP Ronda**					
Pearson r	1	-0.379	0.233	0.438	0.075
P value		**0.046**	0.233	**0.020**	0.705
**FGP Grazalema**					
Pearson r	1	-0.008	0.346	0.371	0.365
P value		0.970	0.090	0.068	0.073
**FGP Los Reales**					
Pearson r	1	-0.132	0.672	0.765	0.513
P value		0.590	**0.002**	**0.000**	**0.025**
**FGP Sierra Real**					
Pearson r	1	-0.009	0.486	0.443	0.401
P value		0.970	**0.035**	0.057	0.089

Significant *P* values are in bold ($P < 0.05$).

Table S5. Significance by three-way ANOVA of single and combined effects of the population, temperature and light for shoot and root length of seedlings obtained from *Abies pinsapo* seeds.

Predictor variable	Shoot length	Root length
Population (P)	**0.000**	0.202
Temperature (T)	**0.000**	**0.000**
Light (L)	0.993	0.850
P x T	0.555	**0.034**
P x L	0.430	**0.002**
T x L	0.489	0.676
P x T x L	0.906	**0.045**

Significant *P* values are in bold ($P < 0.05$).

Table S6. Significance by two-way ANOVA of single and combined effects of the temperature and light for shoot and root length of seedlings obtained from *Abies pinsapo* seeds from different provenances.

Predictor variable	Shoot length	Root length
**Yunquera**		
Temperature (T)	**0.000**	**0.000**
Light (L)	0.981	0.394
T x L	0.506	0.538
**Ronda**		
Temperature (T)	**0.000**	**0.000**
Light (L)	0.430	0.091
T x L	0.730	**0.048**
**Grazalema**		
Temperature (T)	**0.000**	**0.000**

Light (L)	0.100	**0.000**
T x L	0.176	**0.006**
**Los Reales**		
Temperature (T)	**0.001**	**0.008**
Light (L)	0.810	0.299
T x L	0.810	0.338
**Sierra Real**		
Temperature (T)	**0.005**	0.052
Light (L)	0.417	0.080
T x L	0.741	0.705

Significant *P* values are in bold (*P* < 0.05).
